# A High-quality Reference Genome and Tissue Expression Atlas for the European Lobster (*Homarus gammarus*)

**DOI:** 10.1093/gbe/evag138

**Published:** 2026-06-04

**Authors:** Josephine R Paris, Tom L Jenkins, Joan Ferrer Obiol, Manu K Gundappa, Tim Regan, Lahcen Campbell, Gareth L Maslen, Jorge Alvarez-Jarreta, Sarah Dyer, Aaron R Jeffries, Georgina Murray, Audrey Farbos, Lisa K Bickley, Bas Verbruggen, Kelly S Bateman, Carly L Daniels, Charlie D Ellis, Thomas J Ashton, Charles R Tyler, Grant D Stentiford, Ross D Houston, Tim P Bean, Ronny van Aerle, Daniel J Macqueen, Jamie R Stevens, Eduarda M Santos

**Affiliations:** Department of Biosciences, Faculty of Health and Life Sciences, Geoffrey Pope Building, University of Exeter, Exeter, UK; Department of Biosciences, Faculty of Health and Life Sciences, Geoffrey Pope Building, University of Exeter, Exeter, UK; Department of Environmental Science and Policy, University of Milan, Milan, Italy; Department of Biology, Colorado State University, Fort Collins, CO, USA; The Roslin Institute and Royal (Dick) School of Veterinary Studies, University of Edinburgh, Easter Bush Campus, UK; The Roslin Institute and Royal (Dick) School of Veterinary Studies, University of Edinburgh, Easter Bush Campus, UK; European Molecular Biology Laboratory, European Bioinformatics Institute, Cambridge CB10 1SD, UK; European Molecular Biology Laboratory, European Bioinformatics Institute, Cambridge CB10 1SD, UK; European Molecular Biology Laboratory, European Bioinformatics Institute, Cambridge CB10 1SD, UK; European Molecular Biology Laboratory, European Bioinformatics Institute, Cambridge CB10 1SD, UK; Department of Biosciences, Faculty of Health and Life Sciences, Geoffrey Pope Building, University of Exeter, Exeter, UK; Department of Biosciences, Faculty of Health and Life Sciences, Geoffrey Pope Building, University of Exeter, Exeter, UK; Department of Biosciences, Faculty of Health and Life Sciences, Geoffrey Pope Building, University of Exeter, Exeter, UK; Department of Biosciences, Faculty of Health and Life Sciences, Geoffrey Pope Building, University of Exeter, Exeter, UK; Sustainable Aquaculture Futures, University of Exeter, Exeter, UK; Department of Biosciences, Faculty of Health and Life Sciences, Geoffrey Pope Building, University of Exeter, Exeter, UK; Sustainable Aquaculture Futures, University of Exeter, Exeter, UK; Sustainable Aquaculture Futures, University of Exeter, Exeter, UK; Centre for Environment, Fisheries and Aquaculture Sciences (Cefas), Weymouth, UK; The National Lobster Hatchery, Padstow PL28 8BL, UK; Renewable Energy Group, Faculty of Environment, Science, and Economy, University of Exeter, Penryn, UK; Department of Biosciences, Faculty of Health and Life Sciences, Geoffrey Pope Building, University of Exeter, Exeter, UK; The National Lobster Hatchery, Padstow PL28 8BL, UK; Native Aqua Ltd, Dundee DD5 2BG, UK; Department of Biosciences, Faculty of Health and Life Sciences, Geoffrey Pope Building, University of Exeter, Exeter, UK; Sustainable Aquaculture Futures, University of Exeter, Exeter, UK; Sustainable Aquaculture Futures, University of Exeter, Exeter, UK; Centre for Environment, Fisheries and Aquaculture Sciences (Cefas), Weymouth, UK; The Roslin Institute and Royal (Dick) School of Veterinary Studies, University of Edinburgh, Easter Bush Campus, UK; Benchmark Genetics, Edinburgh, UK; The Roslin Institute and Royal (Dick) School of Veterinary Studies, University of Edinburgh, Easter Bush Campus, UK; Sustainable Aquaculture Futures, University of Exeter, Exeter, UK; Centre for Environment, Fisheries and Aquaculture Sciences (Cefas), Weymouth, UK; The Roslin Institute and Royal (Dick) School of Veterinary Studies, University of Edinburgh, Easter Bush Campus, UK; Department of Biosciences, Faculty of Health and Life Sciences, Geoffrey Pope Building, University of Exeter, Exeter, UK; Department of Biosciences, Faculty of Health and Life Sciences, Geoffrey Pope Building, University of Exeter, Exeter, UK; Sustainable Aquaculture Futures, University of Exeter, Exeter, UK

**Keywords:** crustacea, decapod, homarid, immunity, longevity, reference genome

## Abstract

Homarid lobsters are notable for their remarkable immunity and longevity, with lifespans reaching up to 80 years in the wild. A reference genome is available for the American lobster (*Homarus americanus*), but not for the European lobster (*H. gammarus*), despite its ecological significance and importance to fisheries and aquaculture. Here, we present a high-quality genome assembly and annotation for the European lobster. The assembly spans 1.76 Gb, with a scaffold N50 of 1.82 Mb and a BUSCO completeness of 97.6% (96.8% single-copy, 0.8% duplicated). As observed in the American lobster, the total assembly span is substantially smaller than genome size estimates derived from flow cytometry (3.18 to 3.42 Gb). This discrepancy may reflect the highly repetitive nature of decapod genomes, with 51.8% of the European lobster assembly consisting of repetitive elements. Leveraging a comprehensive multi-tissue RNA-seq dataset, we annotated 23,223 protein-coding genes and characterized gene expression across ten tissues to generate a tissue gene expression atlas, available at www.LobsterGeneX.com. Using single-copy orthologues, we estimated a divergence time of 26 Mya between *H. gammarus* and *H. americanus*, corresponding to the Oligocene–Miocene boundary. We also identified lobster-specific gene duplications with roles in immunity and longevity, including telomere maintenance. These genomic resources will facilitate future research into lobster biology, supporting sustainable fisheries and aquaculture, while enabling investigations of homarid evolutionary adaptations.

Significance The European lobster (*Homarus gammarus*) and American lobster (*H. americanus*) are long-lived benthic decapods of high seafood value, typically living 30 to 55 years in the wild, with maximum lifespans reaching up to 80 years. Lobsters grow, reproduce, and regenerate limbs throughout life, with few reports of tumors or age-related disease. We generated a high-quality genome assembly and annotation for the European lobster alongside a tissue expression atlas, LobsterGeneX, which enables the visualization of gene expression profiles across ten tissue types. To demonstrate the value of the genome, we estimated the divergence time between *H. gammarus* and *H. americanus* (26 Mya) and identified duplication events for genes related to immunity and longevity. The European lobster reference genome will facilitate research into local adaptation in lobster populations, hybridization, and the identification of genes of interest in the context of aquaculture, aging, regeneration, disease, and cancer resistance.

## Introduction

Decapods are a diverse and widespread group of crustaceans with important ecosystem roles, often possessing high commercial value (Behringer and Duermit-Moreau 2021). Decapod genomes are notoriously diverse. For instance, genome assembly sizes range from 0.15 Gb (*Pandalus platyceros*) to 5.86 Gb (*Palaemon carinicauda*), and haploid chromosome numbers range from 8 (*Trichoniscus pusillus*) to 104 (*Paralithodes camtschaticus*) (Challis et al. 2023). A particularly notable feature of sequenced decapod genomes is that the assembled genome is typically shorter than estimates derived from *k*-mer profiling and/or flow cytometry. This has been associated with the highly repetitive nature of decapod genomes (Zeng et al. 2025) but may also be due to somatic endopolyploidy and supernumerary B chromosomes (Hughes 1982). These characteristics highlight the complexity of decapod genomes and the importance of high-quality reference assemblies for understanding genome architecture and evolution.

The European lobster (*Homarus gammarus*) is a large decapod of considerable economic and ecological importance. It is a valued seafood species with a long history of fisheries exploitation across the northeast Atlantic and Mediterranean coasts. However, slow growth rates and size-specific fecundity (Ellis et al. 2015a) have increased its vulnerability to overfishing and stock collapse (Kleiven et al. 2022). This has led to fishing restrictions and numerous efforts across Europe to supplement wild stocks with hatchery-reared juveniles (Ellis et al. 2015b; Jenkins et al. 2020). Recent progress in rearing techniques has shown promise for developing lobster aquaculture (Hinchcliffe et al. 2022; Clarke et al. 2023), which may alleviate current fisheries pressures and unlock new markets for lobster trade. European lobsters also have many interesting biological features. For example, they have an average natural lifespan of 30 to 55 years, which can reach 80 years (Sheehy et al. 1999). They continue to grow, reproduce, and regenerate limbs throughout life, showing no observable decline in function (Vogt 2012), with few reports of tumors or ageing-associated diseases, despite their marked longevity (Vogt 2008).

While a reference genome is available for the American lobster (*H. americanus*) (Polinski et al. 2021), no such resource currently exists for the European lobster. Given the biological, fisheries, and aquaculture importance of the species, a reference genome would represent a valuable resource, for example, by facilitating investigations into local adaptation (Jenkins et al. 2019a; Ellis et al. 2023, 2024), for characterizing the genetic basis of interspecific hybridization (Ellis et al. 2020), and aiding the identification of genes associated with desirable traits in aquaculture (Houston et al. 2020; Hinchcliffe et al. 2022). Here, we address this deficit by reporting a high-quality reference genome and annotation for the European lobster. We use the genome and its annotation to (i) quantify gene expression across ten tissue types; (ii) estimate the divergence time between *H. gammarus* and *H. americanus*; and (iii) survey *Homarus* genes linked to immunity and longevity.

## Results and Discussion

### Genome Assembly and Repeat Content

The European lobster assembly consists of 4,133 scaffolds (4,350 contigs) and spans 1.76 Gb, with a scaffold N50 of 1.82 Mb (contig N50 1.6 Mb) and a high BUSCO completeness of 97.6% (96.8% single-copy [S]; 0.8% duplicated [D], with 0.9% fragmented [F], and 1.5% missing [M]). Karyotyping of both *H. gammarus* and *H. americanus* has identified a highly variable number of chromosomes (Hughes 1982). A ploidy analysis (Fig. S1) indicated a diploid genome with most *k*-mer pairs (92%) assigned to an AB configuration with a minor AAAB signal (8%), which may reflect local copy number variation, aneuploidy, or the presence of supernumerary chromosomes.

European lobster meiotic counts vary from *n* = 45 to 88. Taking this as an approximation of the number of chromosomes, ∼16% of the assembly is contained in the largest 45 contigs and ∼25% in the largest 88 contigs. While the assembly is high-quality, future attempts to scaffold contigs into chromosomes would be useful for increasing contiguity and completeness. Nevertheless, the overall contig count (4,350) is >10-fold lower than the American lobster assembly (55,595). For the European lobster, we estimated a *k*-mer quality value (QV) of 38.06 and a *k*-mer completeness of 89.16%. Similarly, the American lobster had a *k*-mer QV of 22.59 but a *k*-mer completeness of only 17.32%. A full comparison between the two *Homarus* assemblies can be found in Table 1.

**Table 1:** Comparison of genome assembly and annotation statistics for the European lobster (*Homarus gammarus*) and American lobster (*Homarus americanus*)

…	European lobster (*Homarus americanus*)	American lobster (*Homarus americanus*)
**Assembly metrics**	…	…
Assembly accession	GCA_958450375	GCF_018991925
Total size (bp)	1,761,786,531	2,292,076,018
Number of contigs	4,350	55,595
Contig N50	1.6 Mb	133.3 Kb
Number of scaffolds	4,133	47,245
Scaffold N50	1.8 Mb	759.6 Kb
Longest scaffold	15.6 Mb	42.5 Mb
GC content	42.50%	43.00%
Repeat content	51.80%	50.60%
BUSCO assessment^a^	C:97.6%[S:96.8%,D:0.8%],F:0.9%,M:1.5%,E:5.6%	C:96.4%[S:95.5%,D:0.9%],F:1.8%,M:1.8%,n:1013,E:5.8%
*k*-mer completeness	89.16%	17.32%
*k*-mer quality	38.06	22.59
**Annotation metrics**	EMBL-EBI Ensembl	Gloucester Marine Institute
Number of protein-coding genes	23,223	22,368
Number of non-coding genes:	26,489	4,513
Small non-coding genes	5,318	2,135
Long non-coding genes	21,031	2,378
Pseudogenes	2	574
Gene transcripts	89,646	47,807
BUSCO assessment^a^	C:97.4%[S:95.6%,D:1.8%],F:2.0%,M:0.6%	C:97.6%[S:96.2%,D:1.4%],F:1.3%,M:1.1%
OMArk assessment^b^	C:92.76%[S:87.98%,D:4.78%],M:7.24%	C:92.03%[S:86.08,D:5.95%],M:7.97%
OMArk lineage placement^c^	C:52.24%[P:18.84%,F:3.81%],I:12.35%[P:7.0%,F:2.17%],U:35.41%	C:57.03%[P:18.06%,F:4.48%],I:14.99%[P:7.24%,F:2.78%],U:27.98%

^a^BUSCO completeness assessment was performed on the arthropoda_odb10 (*n* = 1,013) dataset and comprises complete (C), including single-copy (S) and duplicated (D), fragmented (F), missing (M), and stop codons (errors, E) orthologues (genome only).

^b^OMArk completeness assessment was performed on the Pancrustacea (*n* = 4,103) dataset and comprises complete (C), including single-copy (S), duplicated (D), and missing (M) Hierarchical Orthologous Groups (HOGs).

^c^OMArk lineage assessment was performed on the Pancrustacea (*n* = 4,103) dataset and comprises consistent (C) lineage placement, including partial (P) and fragmented (F) hits, and inconsistent (I) lineage placement, including partial (P) and fragmented (F) hits, and unknown (U) lineage placement.

To illustrate the European lobster assembly's utility, we examined the *Dscam* gene, a member of the immunoglobulin superfamily with roles in neural development and immune function. In arthropods, *Dscam* produces multiple pathogen-specific receptors via immune-responsive alternative splicing (Li et al. 2022). In the American lobster assembly, *Dscam* was fragmented across two scaffolds (Polinski et al. 2021). By contrast, we identified a single, intact *Dscam* locus (ENSHGAG00000010168) on scaffold 3867 (Fig. S2), encoding a full protein of 1,139 amino acids. *Dscam* expression was detected across all tissues, with the highest expression in neural tissue, followed by the heart (Fig. S3).

**Fig. 1.**
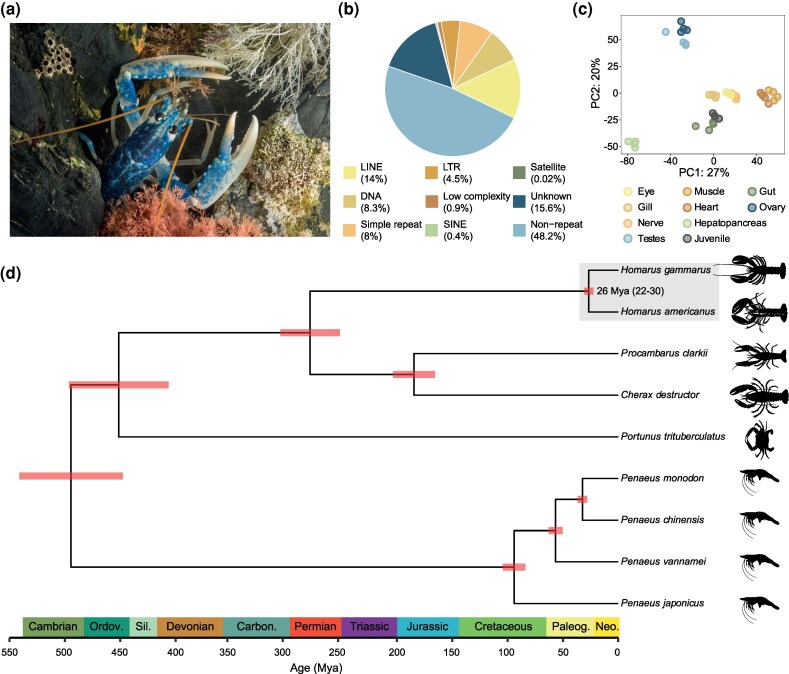
Genome assembly and annotation of the European lobster, Homarus gammarus (a) photograph of a juvenile European lobster (photograph credit: Alex Hyde). (b) Characterization of the transposable element (TE) landscape, showing that >50% of the genome comprises repetitive elements. (c) Principal Component Analysis (PCA) of normalized gene expression counts across the 10 tissues used in the LobsterGeneX gene expression atlas. (d) Divergence dating between the European lobster (Homarus gammarus) and the American lobster (Homarus americanus). BEAST time-tree of nine selected decapod species generated using 25 clock-like orthologues. The gray box highlights the estimated divergence time between H. gammarus and H. americanus (26 Mya; 95% HPD: 22 to 30 Mya) and the red horizontal node bars show the 95% HPD for node ages.

Genome size estimation using *k-*mer profiling produced an estimate of 2.04 Gb, decreasing to 1.80 Gb after reducing the number of repeat *k-*mers included in the estimate, consistent with the assembly span. Indeed, 51.8% of the assembly is repetitive (Fig. 1b), similar to American lobster (50.6% repeat content). Total repeat content and transposable element (TE) families were very similar between the two species (Table S1), consistent with the moderate TE diversity observed across crustaceans (Zeng et al. 2025). Flow cytometry estimated the *H. gammarus* genome at 3.18 to 3.42 Gb, while estimates for *H. americanus* are 4.30 to 4.65 Gb, nearly double the assembly size in both species. The lower *k*-mer completeness suggests that complex repetitive landscapes have led to missing or collapsed regions in both *Homarus* assemblies.

### Genome Annotation

We generated a comprehensive RNA-seq dataset from ten tissues (eye, gill, gut, heart, hepatopancreas, juvenile, muscle, nerve, ovary, testes). The EMBL-EBI Ensembl Gene Annotation pipeline predicted 23,223 protein-coding genes. BUSCO and OMArk analysis revealed high annotation completeness (BUSCO: 97.4% [95.6% single-copy, 1.8% duplicated]; OMArk: 92.76%). Compared to the American lobster annotation, we identified a higher number of long non-coding RNAs (lncRNAs). While many may be genuine, this apparent inflation may reflect technical artifacts, highlighting the need for standardized annotation methods for comparative studies (Prieto-Baños et al. 2025).

### Tissue-expression Atlas for the European Lobster

To increase the value of this genome resource to the research community, we developed a tissue expression atlas for the 23,223 annotated protein-coding genes. The atlas (LobsterGeneX; available at www.LobsterGeneX.com) is an interactive application that allows users to select a gene and visualize its expression across nine tissue types and juvenile whole-body samples, each with four biological replicates. This open resource will enhance future studies aiming to characterize the expression of lobster genes, including those related to immunity and longevity, and the optimization of hatchery and aquaculture practices.

### Divergence Time Between *H. gammarus* and *H. americanus*

A phylogeny built using 1,295 orthologues from nine decapod species showed the expected evolutionary relationships among the included species. Using the 25 most clock-like single-copy orthologues and three calibration points, we produced a time-calibrated phylogeny and inferred a divergence time of 26 Mya (95% highest posterior density interval: 22 to 30 Mya) between *H. gammarus* and *H. americanus* (Fig. 1d). Results were robust to different clock models and changes in the prior distributions of calibration points (Table S2). Our divergence time is markedly more recent than a previous estimate of 100.9 Mya derived from three mitochondrial genes and three nuclear genes (Bracken-Grissom et al. 2014), but older than an earlier published report of 18 Mya using one mitochondrial and three nuclear genes (Porter et al. 2005). Broader genome-wide sampling is expected to provide more robust divergence time estimates than analyses based on a smaller number of loci.

### Gene Duplications in *Homarus*

In comparison to seven other decapods, we identified eight simple duplications (Table S3) and 80 complex duplications (Table S4) in the *Homarus* branch (Fig. S4). Simple gene duplications are single-copy in other decapods but duplicated in both *Homarus* species, whereas complex duplications involve multicopy gene families further expanded in Homarus (see Methods).

We identified a pronounced expansion of ligand-gated ion channels (LGICs), which mediate chemical signal transmission to cells, particularly neurons. Together with three complex duplications in other neurofunction-related gene families, this expansion suggests *Homarus* lobsters have evolved enhanced chemosensory mechanisms for benthic living (Derby and Weissburg 2014).

We also detected expansions in immune-related gene families, including pentaxins, an evolutionarily conserved family involved in acute immune responses, as well as immunoglobulin domains, which play key roles in immune recognition. C-type lectins (identified as a simple duplication) were also expanded; these proteins are widely implicated in innate and adaptive antimicrobial defense (Brown et al. 2018). Although such gene families are often dynamic across animal lineages, their expansion in *Homarus* highlights candidate loci for future investigation of lobster immunity, ecology, and evolution.

Finally, we identified four complex duplications in gene families associated with genome organization, alongside several simple and complex duplications with putative roles in telomere maintenance. These included: (i) reverse transcriptase (RNA-dependent DNA polymerase), present as six and seven copies in *H. gammarus* and *H. americanus*, respectively; and (ii) telomerase-activating protein *est1*, identified as a simple duplication in *Homarus*, with both copies expressed across all *H. gammarus* tissues (Fig. S4). Elevated telomerase activity has been reported across multiple lobster tissues (Klapper et al. 1998), consistent with a role in sustaining long-term cell proliferation and limiting senescence. Expansion of telomere-associated gene families may therefore contribute to this high telomerase activity and potentially, to longevity. We also detected expansions in gene families linked to molting, including chitin-related proteins, suggesting increased diversity of growth-associated genes in *Homarus* relative to other decapods.

Simple duplications, including those involving C-type lectin domains and telomerase-activating protein *est1* are candidates for lineage-specific duplication events that underwent functional divergence or neofunctionalization during *Homarus* evolution. Comparative analyses of these paralogues could test whether they have acquired distinct functional specializations, providing a framework to evaluate the adaptive significance of gene duplications unique to this lineage.

## Conclusions

We present the first high-quality reference genome and annotation for the European lobster, together with a multi-tissue expression atlas to support genotype–phenotype studies. Although highly complete, the assembly is shorter than flow cytometry estimates, reflecting the repeat-rich nature of *Homarus* genomes and the need for improved repeat resolution and chromosome-scale scaffolding. Using this new resource, we dated the speciation event separating *H. gammarus* and *H. americanus* and identified lobster-specific gene family expansions linked to neurofunction, immunity, and telomere biology, providing a foundation for studies of adaptation, longevity, and fisheries-relevant traits.

## Materials and Methods

### Genome Sampling, DNA Extraction and Sequencing

Tissue was obtained from a wild adult male (5 to 7 years old; carapace length >87 mm) collected in Cornwall, UK. Muscle from the crusher claw was excised, flash-frozen and stored at −80 °C. HMW DNA was extracted using a modified salting-out protocol developed for lobster pleopods (Jenkins et al. 2019b). DNA was cleaned with modifications aimed at reducing DNA shearing (see Supplementary Materials).

Libraries were prepared using the Pacific Biosciences SMRTbell Express Template Preparation Kit. Size selection was performed on a BluePippin at a >15 Kb cut-off threshold. Long-read sequencing was performed on a PacBio Sequel across 14 SMRT Cells (1M v3 and Sequel Sequencing Chemistry v3), generating 106 Gb of data (∼60× coverage). Using DNA obtained from the same sample, we performed Illumina sequencing (600 Gb of data, ∼300× coverage) on two lanes of an Illumina NovaSeq S1 flow cell (150 bp paired-end). Data were cleaned using fastp v0.23.4 (Chen et al. 2018).

### RNA Sampling, Extraction and Sequencing

We generated a comprehensive RNA-seq dataset from nine tissues (eye, gill, gut, heart, hepatopancreas, muscle, nerve, ovary, testes) together with whole juvenile samples. Further details on sampling can be found in the Supplementary Materials.

Samples were disrupted by grinding frozen tissue with liquid nitrogen before homogenization in lysis reagent. RNA was extracted using the Qiagen mRNeasy Mini Kit, including an on-column DNase digestion step. RNA quality was quantified using an Agilent 2100 Bioanalyzer with RNA 6000 nano kit. mRNA isolation was performed via poly(A) enrichment using the Illumina Tru-Seq Low Throughput protocol. cDNA libraries were constructed using Illumina's TruSeq Stranded mRNA Sample Preparation kit. Libraries were sequenced on an Illumina HiSeq 2500 (100 bp paired-end).

### Genome Assembly

Genome size was estimated by *k*-mer profiling and flow cytometry. *k*-mers (size 21) were counted using dsk v2.3.3 (Rizk et al. 2013)*. k*-mer profiles were assessed for genome size, heterozygosity and duplication rate in GenomeScope v2 (Ranallo-Benavidez et al. 2020). To assess the impact of repetitive sequences on genome size estimation, *k*-mers were summarized using coverage threshold cut-offs of 100,000 and 1,000,000. smudgeplot v0.2.3 was used to assess ploidy using 21 and 1600 for the lower and upper cutoffs, respectively.

Flow cytometry was performed on eye tissue and two replicates of hepatopancreas derived from a single adult individual sampled from St Andrews, UK. Mean nuclear fluorescence was measured on a Merck Millipore Muse Cell Analyzer flow cytometer (532 nm laser). Further details can be found in the Supplementary Materials.

Flye v2.8.1 (Kolmogorov et al. 2019) was used to assemble the long-read data. The optimum assembly (based on N50 and BUSCO completeness) was generated using: (i) minimum read length of 1000 bp, (ii) –minimum-overlap 10000, and (iii) –asm-coverage 40. The assembly was polished with short-read data using NextPolish v1.4.0 (Hu et al. 2019). Quality assessment after two rounds of polishing showed no improvement over the first round, so only one polishing iteration was applied. Potential contamination in the assembly was assessed using BlobTools v1.1.1 (Laetsch and Blaxter 2017), identifying one potential contig as a contaminant, which was removed from the assembly. Haplotype purging was performed using purge_dups v1.2.5 (Guan et al. 2020). Genome completeness was assessed using BUSCO v5.7.1 (Manni et al. 2021) against the arthropoda_odb10 dataset (*n* = 1013). Merqury v1.3 (Rhie et al. 2020) was used to perform a *k*-mer assessment of quality and completeness using the short-read data (*k*-mer = 21).

### Genome Annotation

Structural annotations were generated via the EMBL-EBI Ensembl Gene Annotation workflow (Aken et al. 2016). Following the Ensembl core database schema, the assembly was first screened for repetitive sequences using RepeatMasker v4.1.0 (Smit et al. 2015). A de novo repeat library was generated using RepeatModeller v2.0.1 (Flynn et al. 2020). Because RepeatModeller libraries may contain non-TE protein coding sequences, we opted to perform repeat library filtration of TEs using proteins from *H. americanus* (GCF_018991925.1) to avoid potentially over-masking protein coding loci with genuine TE-protein overlaps. After selecting the longest isoform per gene, the structural annotation was assessed using both BUSCO v5.7.1 (Manni et al. 2021) (arthropoda_odb10, *n* = 1,013) and OMArk v2.0.3 (Nevers et al. 2025) (Pancrustacea, *n* = 4,103).

### Gene Expression Atlas

For the tissue expression atlas, RNA-seq reads were aligned to the genome using STAR v2.7.3 (Dobin et al. 2012). Alignments were used as input to featureCounts (Liao et al. 2014), only counting gene features where both paired-end reads aligned. Normalized counts and the variance stabilizing function (VST) were computed using DESeq2 (Love et al. 2014).

### Orthogroup Identification

We searched for decapod genomes using GoaT (Challis et al. 2023). The quality of 21 proteomes was assessed for completeness with BUSCO (Manni et al. 2021) and the level of duplication with CD-hit v4.81 (Fu et al. 2012). To account for potential biases introduced by the different annotation pipelines used for *H. gammarus* and *H. americanus* (Prieto-Baños et al. 2025; Table 1), we performed an annotation of *H. americanus* using the EMBL-EBI Ensembl Gene Annotation workflow. After quality assessment of the 21 proteomes, we retained nine species for downstream analyses: *H. gammarus*, *H. americanus*, *Cherax destructor*, *Penaeus chinensis*, *Penaeus japonicus*, *Penaeus monodon*, *Penaeus vannamei*, *Portunus trituberculatus*, and *Procambarus clarkii*. We also included quality-filtered proteomes for three outgroups: *Daphnia magna*, *Drosophila melanogaster* and *Hyalella azteca*.

Following previous approaches (Regan et al. 2021), proteomes were clustered into orthogroups using OrthoFinder v2.5.4 (Emms and Kelly 2019). OrthoFinder was run using Diamond v2.0.15 (Buchfink et al. 2021) for clustering, MUSCLE v3.8.1551 (Edgar 2004) for Multiple Sequence Alignment (MSA) and RAxML-NG v0.9.0 (Kozlov et al. 2019) for tree inference of the MSAs. Overall, 89.4% of genes were assigned to an orthogroup and 12.1% of orthogroups were species-specific.

### Divergence Dating

We estimated the divergence time between *H. gammarus* and *H. americanus* using phylogenetic analyses with molecular dating. Taxon sampling was restricted to the nine decapod species used for orthogroup identification. A concatenated dataset of 1,295 single-copy orthologues was used to estimate a maximum-likelihood phylogeny using RAxML-ng v1.1.0 (Kozlov et al. 2019). Following this, 25 of the most clock-like single-copy orthologues were used to conduct divergence time analysis in BEAST v2.6.7 (Bouckaert et al. 2019) using three calibration points (Table S5). Calibration points were based on fossil-calibrated divergence estimates from taxon-rich datasets (Ma et al. 2009; Bracken-Grissom et al. 2014; Wolfe et al. 2019). Further details can be found in the Supplementary Materials.

### Identification of Gene Duplications and Expansions in *Homarus*

To identify gene duplications specific to the *Homarus* branch, we compared the two *Homarus* species against seven decapod species and three outgroups. We grouped *Homarus*-specific duplications into two categories: (i) simple duplications (a single duplication event in the *Homarus* branch), and (ii) multi-copy expansions (orthogroups showing a fold change >2.0 and a Mann–Whitney *U P*-value ≤0.05 when compared to the mean of all other decapods).

We identified an initial set of 20 simple duplications, which were reduced to eight candidates for analysis after performing quality checks to reduce the incorrect inference of split-gene annotations (i.e. cases where a single gene is incorrectly annotated as two or more separate models) as gene duplicates (see Supplementary Materials).

To explore the functional annotation of duplicated genes, we performed an analysis of the protein domains using InterProScan v.5 (Jones et al. 2014), using the top Pfam domain for functional inference. For multi-copy expansions, orthogroups lacking Pfam domains or present in fewer than five of seven Decapoda species were excluded, resulting in a final set of 80 duplicated orthogroups.

## Supplementary Material

evag138_Supplementary_Data

## Data Availability

Raw sequence data are available under the BioProject accession PRJEB63315. The *Homarus gammarus* genome assembly is available on NCBI and the ENA under the accession GCA_958450375.1. The Ensembl Metazoa annotation for *Homarus gammarus* is available at: https://metazoa.ensembl.org/Homarus_gammarus_gca958450375v1/. The Ensembl Metazoa annotation for *Homarus americanus* is available at: https://beta.ensembl.org/species/efd4e835-043c-4e17-917d-ef88bc7475e6. The assembly is also available at the UCSC Genome Browser: https://genome.ucsc.edu/cgi-bin/hgTracks?genome=GCA_958450375.1&hubUrl=/gbdb/genark/GCA/958/450/375/GCA_958450375.1/hub.txt. LobsterGeneX is freely available for use at www.LobsterGeneX.com. Expression matrices are available on the LobsterGeneX GitHub: https://github.com/Tom-Jenkins/LobsterGeneX/tree/main/public, which is archived under Zenodo DOI: https://doi.org/10.5281/zenodo.17233964.
