# Supplementary material for: A High-quality Reference Genome and Tissue Expression Atlas for the European Lobster (Homarus gammarus)
Source: Genome Biol Evol. 2026 Jun 4;18(6):evag138. doi: 10.1093/gbe/evag138 (PMC13286009; doi:10.1093/gbe/evag138)
Supplement: evag138_Supplementary_Data [file evag138_supplementary_data.zip › supp-2.docx]

**Supplementary Material for**

A high-quality reference genome and tissue expression atlas for the European lobster (*Homarus gammarus*)

Josephine R Paris, Tom L Jenkins, Joan Ferrer Obiol, Manu K Gundappa, Tim Regan, Lahcen Campbell, Gareth L Maslen, Jorge Alvarez-Jarreta, Sarah Dyer, Aaron R Jeffries, Georgina Murray, Audrey Farbos, Lisa K Bickley, Bas Verbruggen, Kelly S Bateman, Carly L Daniels, Charlie D Ellis, Thomas J Ashton, Charles R Tyler, Grant D Stentiford, Ross Houston, Tim P Bean, Ronny van Aerle, Daniel J Macqueen, Jamie R Stevens, Eduarda M Santos

**This supplement contains:**

- Supplementary Methods
- Supplementary Figures S1-S4
- References

**Supplementary Methods**

**Modifications to reduce DNA shearing**

DNA was cleaned using the DNeasy PowerClean CleanUp kit (Qiagen, Germany) with the following modifications: 30 μl of proteinase K was added to the tissue during the cell lysis step and incubated overnight in a thermomixer; for steps where vortexing was required, this approach was replaced by gently inverting samples 50 times, while precipitation of DNA using PowerClean columns was replaced with an isopropanol precipitation method.

**Samples used for flow cytometry calibration**

Samples were calibrated against nuclei derived from European plaice (*Pleuronectes platessa*, 0.4 pg DNA diploid), European flat oyster (*Ostrea edulis*, 1.2 pg DNA diploid), giant tiger prawn (*Penaeus monodon*, 2.5 pg DNA diploid), and rainbow trout (*Oncorhynchus mykiss*, 2.5 pg DNA diploid, 3.5 pg DNA triploid). Genome size was calculated as the ratio of mean fluorescence of sample/standard, multiplied by the known amount of DNA in the standard.

**Samples used for RNA-sequencing**

Four individual adult (two male and two female) individuals originating from wild populations collected from Weymouth, UK, were sampled for eye, gill, gut, heart, hepatopancreas, muscle, and nerve. Four individual adult females were sampled for ovary tissue, and four individual adult males were sampled for testes tissue. Four juvenile lobsters (moult stage 4, unknown sex) were obtained from the National Lobster Hatchery, Cornwall, UK. All samples were flash-frozen and stored at -80^o^C.

**Divergence dating**

To infer the divergence time between *H. gammarus* and *H. americanus*, we performed phylogenetic and divergence time estimation analyses. Taxon sampling was restricted to the nine decapod species used for orthogroup identification. Single-copy orthologs were aligned using MAFFT v7.505 (Katoh & Standley 2013). Poorly aligned regions were trimmed using trimAL v1.4.1 (Capella-Gutiérrez et al. 2009). A concatenated dataset of all 1295 single-copy orthologs was generated using AMAS (Borowiec 2016) and this was subsequently used to estimate a maximum-likelihood (ML) phylogeny using RAxML-ng v1.1.0 (Kozlov et al. 2019). We conducted 50 ML tree searches using 25 random and 25 parsimony-based starting trees using the LG+G4 amino acid substitution model. Following ML tree searches, we generated 500 nonparametric bootstrap replicates and checked for convergence post hoc using the --bsconverge command in RAxML-ng with a cutoff value of 0.03; we used the RAxML-ng --support command to compute bootstrap branch support values.

We used three calibration points: (1) at the root of the phylogeny (Crown Decapoda), (2) at Crown Astacidea, and (3) at the most recent common ancestor (MRCA) of *Penaeus vannamei*, *Penaeus monodon* and *Penaeus chinensis*. Calibration points were based on the posterior distributions of previous divergence time estimation analyses using fossil calibrations derived from more taxon-rich datasets (Bracken-Grissom et al. 2014; Ma et al. 2009; Wolfe et al. 2019). To minimise the effects of among-lineage rate variation, we subsampled the 25 most clock-like single-copy orthologs using SortaDate (Smith et al., 2018). We conducted three divergence time analyses in BEAST v2.6.7 (Bouckaert et al. 2019): (i) using a strict clock model and uniform priors for calibration points, (ii) using a strict clock model and normally distributed priors for calibration points, and (iii) using an uncorrelated log-normal relaxed clock model and normally distributed priors for calibration points.

For each analysis, we executed three independent Markov chain Monte Carlo (MCMC) runs with different starting seed numbers. For each MCMC run, the best-fitted amino acid substitution model as inferred by ModelTest-NG (Darriba et al. 2019) was set for each partition, tree topology and clock model were linked by locus, and we set a Calibrated Yule model (Heled & Drummond 2012) as a tree prior. Each chain was run for 20 million generations, sampling every 20,000 generations and we checked each run for convergence of parameter values and age estimates by inspecting traces and effective sample sizes (ESS) in Tracer v1.7.1 (Rambaut et al. 2018). We then combined tree and log files from each set of three independent runs using LogCombiner v2.6.7 (Bouckaert et al. 2019) and generated maximum-clade-credibility trees with node heights set to mean age estimates discarding the first 10% of each MCMC as burn-in with TreeAnnotator v2.6.0 (Bouckaert et al. 2019). To assess the level of rate heterogeneity across lineages, we examined the coefficient of variation in clock rates. We also generated joint prior distributions for each of the three analyses by running MCMCs with no data.

**Quality checks to reduce the incorrect inference of split-gene annotations**

Protein sequences for each species for each orthogroup were aligned using MAFFT v.7.515 (Katoh & Standley 2013) and cleaned and trimmed using trimAl v.1.4.1 (Capella-Gutiérrez et al. 2009) for visual inspection in AliView v.1.27 (Larsson 2014). Twelve candidate duplications were highly indicative of split-gene models and were removed, resulting in eight duplicated orthogroups for downstream analysis.

**Supplementary Figures**

#
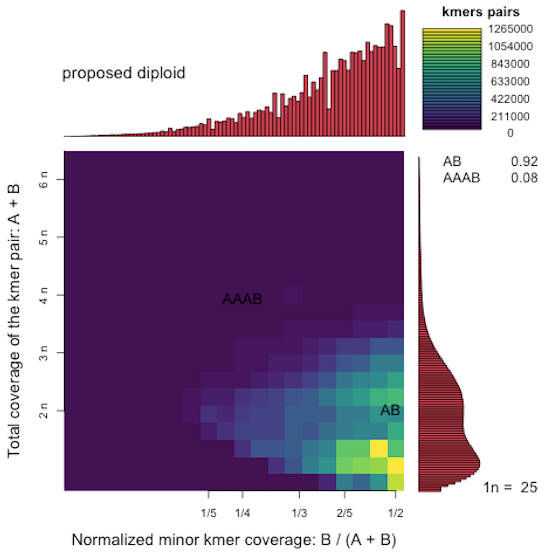


#

#

#

#

#

#

#

# **Figure S1.** smudgeplot analysis of ploidy levels. The European lobster genome is a proposed diploid, with 92% of the *k*-mers showing an AB configuration and 8% showing an AAAB configuration.

# **
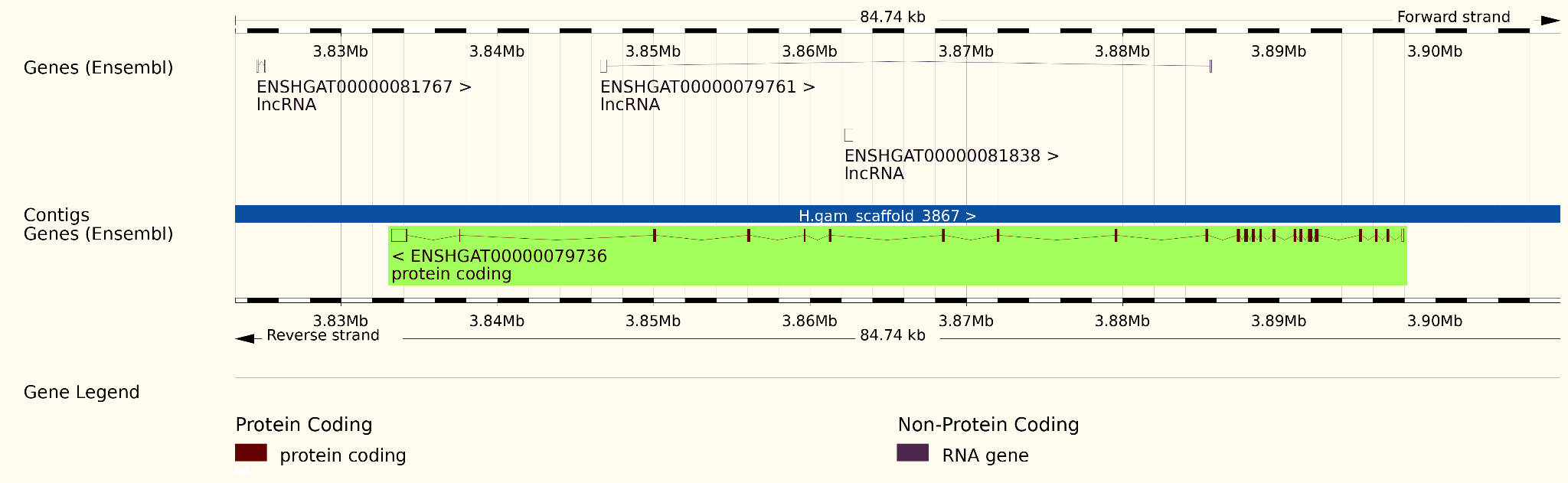
Figure S2.** Characterisation of the *Dscam* gene ([ENSHGAG00000010168](https://metazoa.ensembl.org/Homarus_gammarus_gca958450375v1/Gene/Summary?g=ENSHGAG00000010168;r=H.gam_scaffold_3867:3833226-3897963;t=ENSHGAT00000079736)) and of the transcript ENSHGAT00000079736 on scaffold_3867. This transcript has the Pfam domain: Down syndrome cell adhesion molecule (*Dscam*) C terminal.

#

#
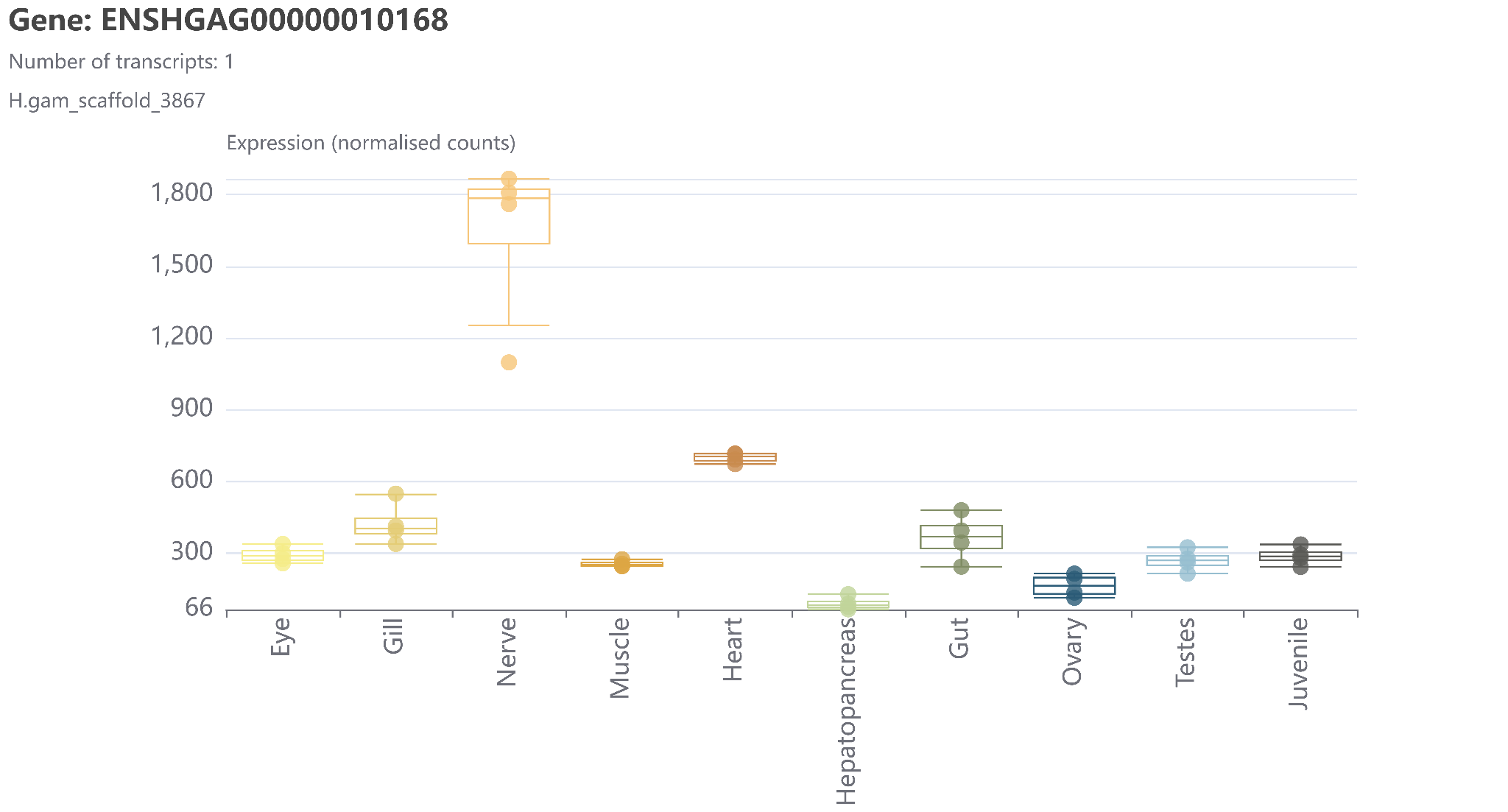
**Figure S3.** Tissue expression profiles in the [ENSHGAG00000010168](http://enshgag00000010168) gene, generated using LobsterGeneX. This gene contains a transcript, ENSHGAT00000079736, that has the Pfam domain: Down syndrome cell adhesion molecule (*Dscam*) C terminal.


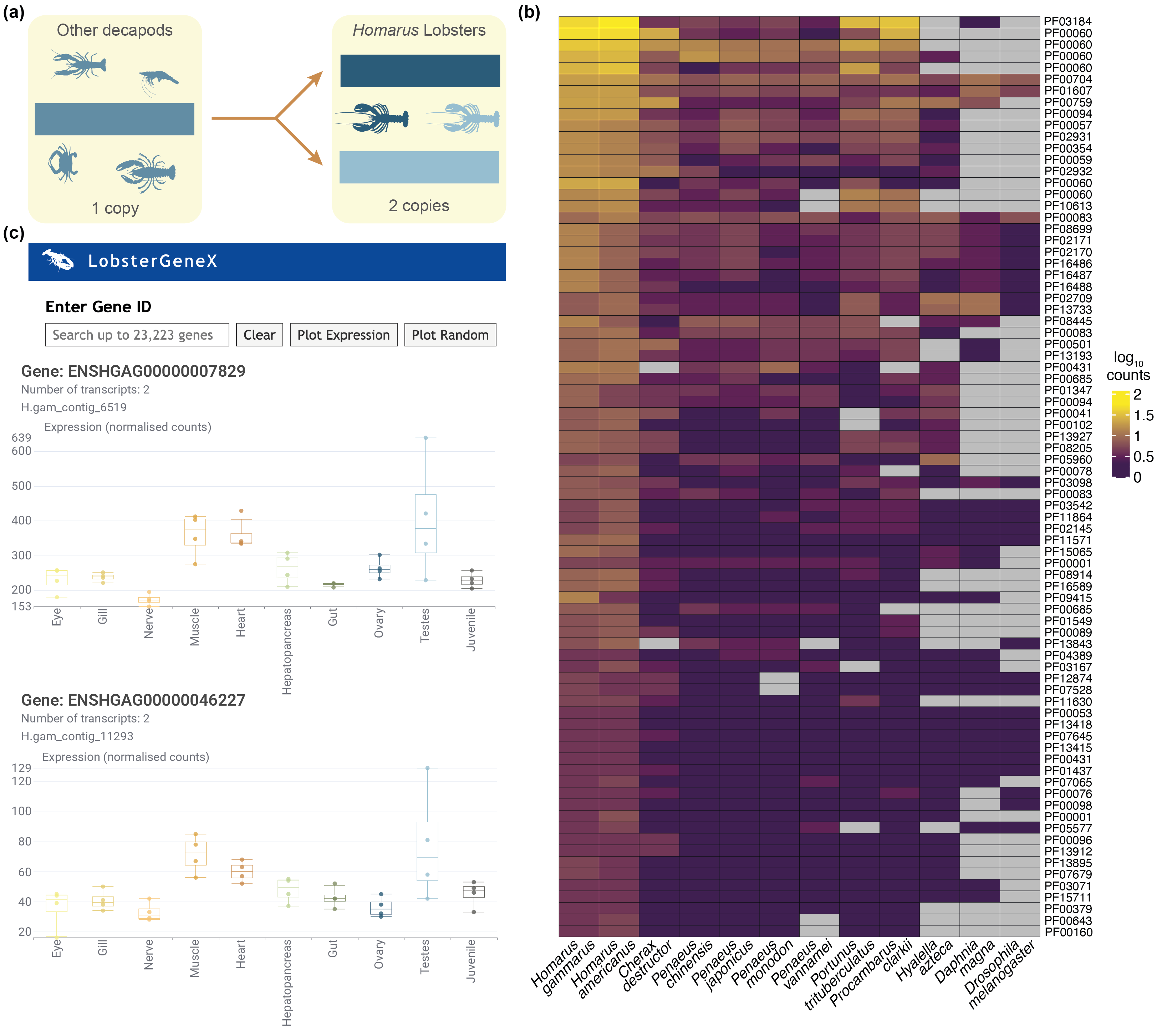


**Figure S4.** Gene duplications in *Homarus*. (**a**) Schematic representation of identification of the simple duplications unique to the *Homarus* branch. (**b**) Heatmap showing the log_10_ normalised counts of the complex duplications across the two *Homarus* species, the European lobster (*Homarus gammarus*) and the American lobster (*Homarus americanus*) compared to the nine other decapod species and three outgroups. (**c**) Visualisation of the gene expression of the *est1* simple duplication (gene IDs: ENSHGAG0000007829 and ​​ENSHGAG0000046227) in LobsterGeneX. These genes have the Pfam domains PF10373 & PF10374, which have the Pfam descriptions: *est1* DNA/RNA binding domain and Telomerase activating protein *est1*, respectively.

**References**

1. Katoh K, Standley DM. 2013. MAFFT multiple sequence alignment software version 7: improvements in performance and usability. Mol. Biol. Evol. 30:772–780.
2. Capella-Gutiérrez S, Silla-Martínez JM, Gabaldón T. 2009. trimAl: a tool for automated alignment trimming in large-scale phylogenetic analyses. Bioinformatics. 25:1972–1973.
3. Borowiec ML. 2016. AMAS: a fast tool for alignment manipulation and computing of summary statistics. PeerJ, 4:e1660.
4. Kozlov AM, Darriba D, Flouri T, Morel B, Stamatakis A. 2019. RAxML-NG: a fast, scalable and user-friendly tool for maximum likelihood phylogenetic inference. Bioinformatics. 35:4453–4455.
5. Bracken-Grissom HD, Ahyong ST, Wilkinson RD, Feldmann RM, Schweitzer CE, Breinholt JW, Bendall M, Palero F, Chan TY, Felder DL, Robles R. 2014. The emergence of lobsters: phylogenetic relationships, morphological evolution and divergence time comparisons of an ancient group (Decapoda: Achelata, Astacidea, Glypheidea, Polychelida). Systematic Biology, 63(4):457-79.
6. Ma KY, Chan T-Y, Chu KH. 2009. Phylogeny of penaeoid shrimps (Decapoda: Penaeoidea) inferred from nuclear protein-coding genes. Mol. Phylogenet. Evol. 53:45–55.
7. Wolfe JM, Breinholt JW, Crandall KA, Lemmon AR, Lemmon EM, Timm LE, Siddall ME, Bracken-Grissom HD. 2019. A phylogenomic framework, evolutionary timeline and genomic resources for comparative studies of decapod crustaceans. Proc. Biol. Sci. 286:20190079.
8. Smith SA, Brown JW, Walker JF (2018) So many genes, so little time: A practical approach to divergence-time estimation in the genomic era. PLOS ONE 13(5): e0197433.
9. Bouckaert R, Vaughan TG, Barido-Sottani J, Duchêne S, Fourment M, Gavryushkina A, Heled J, Jones G, Kühnert D, De Maio N, Matschiner M. 2019. BEAST 2.5: An advanced software platform for Bayesian evolutionary analysis. PLoS computational biology,15(4):e1006650.
10. Darriba D, Posada D, Kozlov AM, Stamatakis A, Morel B, Flouri T. 2020. ModelTest-NG: A new and scalable tool for the selection of DNA and protein evolutionary models. Molecular Biology and Evolution, 37(1): 291-294.
11. Heled J, Drummond AJ. 2012. Calibrated tree priors for relaxed phylogenetics and divergence time estimation. Syst. Biol. 61:138–149.
12. Rambaut A, Drummond AJ, Xie D, Baele G, Suchard MA. 2018. Posterior summarization in Bayesian phylogenetics using tracer 1.7. Syst. Biol. 67:901–904.
13. Katoh K, Standley DM. 2013. MAFFT multiple sequence alignment software version 7: improvements in performance and usability. Mol. Biol. Evol. 30:772–780.
14. Larsson A. 2014. AliView: a fast and lightweight alignment viewer and editor for large datasets. Bioinformatics. 30:3276–3278.
